# Approaches used to monitor the effectiveness of community‐led monitoring programmes: a scoping review to inform HIV programmes

**DOI:** 10.1002/jia2.70020

**Published:** 2025-07-25

**Authors:** Farihah Malik, Nonna Turusbekova, Susan Perez

**Affiliations:** ^1^ University Institute of Public Health The University of Lahore Lahore Pakistan; ^2^ Independent Consultant Antwerp Belgium; ^3^ AIDS Strategy Advocacy and Policy Hanoi Vietnam

**Keywords:** accountability mechanisms, community advocacy, community‐led monitoring, evaluation, HIV, monitoring

## Abstract

**Introduction:**

Community‐led monitoring (CLM) for HIV is a technique implemented by local community‐led organizations and groups that systematically gather data about HIV services to advocate for improvement. This review was conducted to explore fields other than HIV where CLM or similar approaches have been used, and to identify methods and tools used to monitor the effectiveness of such approaches.

**Methods:**

Using a systematic search in PubMed®, Embase® and Web of Science™, we identified publications describing community involvement in the monitoring of public services. We searched for English‐language, peer‐reviewed articles and abstracts published from inception until 7 March 2024 with search terms covering two broad areas: “community‐led monitoring” and “impact/effectiveness.” We double‐screened titles and abstracts and single‐extracted data on publication type, region and geographic location, field, programme goals, the methods used to monitor the programme, indicators used for monitoring and the frequency with which the programme was monitored. In addition, a web search was conducted to identify relevant grey literature.

**Results:**

We identified 282 records, of which 28 publications were included. Additionally, 24 documents were included through a search of grey literature. Seven peer‐reviewed publications related to HIV CLM, 10 were from other health services and 11 were from monitoring of natural resources. No peer‐reviewed publications documented results from routine evaluations of CLM programmes or described a monitoring framework for CLM.

Common themes identified across different fields were the role of multi‐stakeholder collaboration as an enabling factor for community monitoring, challenges in sustainability due to fragmented funding and the inability of existing evaluation approaches to capture the longer‐term impact of community monitoring.

**Discussion:**

Having a robust monitoring and evaluation system is essential for improving CLM programme operations and demonstrating impact. However, demonstrating the impact of community‐led advocacy efforts is complex and more research is needed to assess longer‐term impacts. Monitoring of locally led adaptation programmes for climate resilience offers useful examples of impact assessments.

**Conclusions:**

The synthesized findings and lessons from this scoping review have been used, along with consultations with CLM implementers, to develop a guide to monitor outcomes and impact of HIV CLM programmes.

## 1 INTRODUCTION

**Figure 1:**
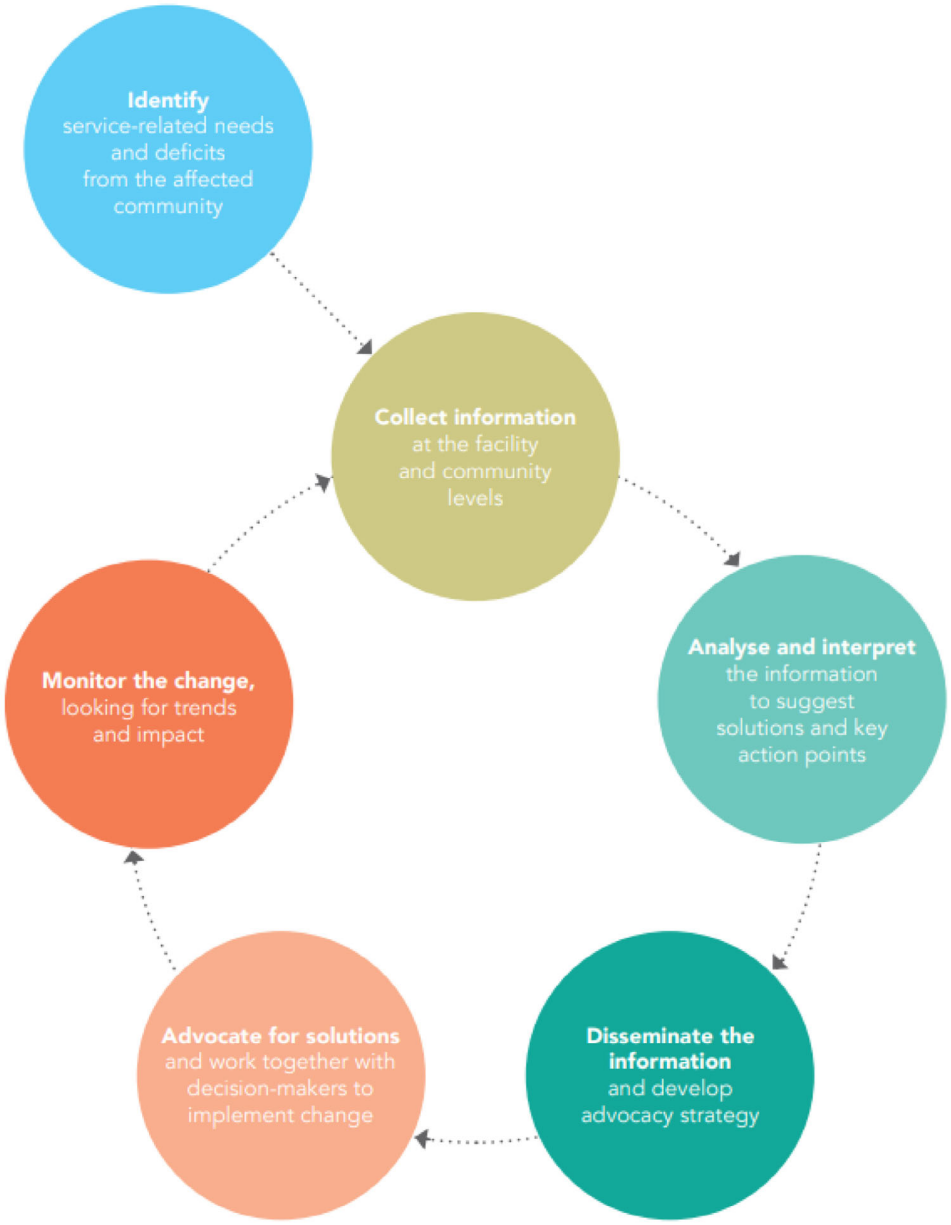
Schematic of the CLM cycle (Source: UNAIDS [5]).

Community‐led monitoring (CLM) is a methodology which improves the quality and effectiveness of public services and empowers affected communities. CLM for health programmes involves communities affected by health inequities, particularly HIV, tuberculosis (TB) and malaria, to actively participate in monitoring and then advocating for improved health services [1, 2]. At its core, CLM involves local community‐led organizations, networks of key populations and other affected groups systematically monitoring health services [1, 2, 3]. By deciding what indicators to monitor, gathering data, analysing it, jointly looking for solutions and advocating for evidence‐driven improvements, CLM works to ensure that health services are responsive to community priorities and experiences (Figure 1). Community leadership also plays a crucial role in combating stigma and discrimination and ensuring that health programmes are evidence‐based and rights‐focused [4].

CLM is often hailed as a new approach, but it is essential to recognize that community action has always been at the forefront of the HIV response [6, 7]. While CLM introduces structured methodologies and data‐driven approaches, it stands on the shoulders of established community advocacy [6, 8]. Nonetheless, health services monitoring where communities take the lead is an evolving field, and its contributions are yet to be sufficiently acknowledged and optimized [9].

While CLM programmes collect rich data on barriers to equitable health service delivery, many CLM implementers face challenges in documenting evidence of programme outcomes and impact to funders and other stakeholders [10, 11]. One challenge is the short timeframe in which funders expect CLM programmes to deliver evidence of impact on health services, policies, community experiences or other outcomes [12]. Another challenge, inherent to advocacy work, is that a successful CLM programme that gathers, analyses and presents data to decision‐makers cannot control whether action is triggered [13]. If no steps are undertaken by decision‐makers, the programme may be judged less successful; if action is taken, the causal contribution of the CLM programme may be difficult to establish.

A robust Monitoring, Evaluation, Accountability, and Learning (MEAL) framework for CLM could address these challenges by enabling regular performance tracking, programme self‐evaluation of effectiveness for continuous improvement and assessment of impact. The main objective of this scoping review was to inform the development of a MEAL framework for CLM, primarily for HIV, based on lessons learnt from CLM for other health areas and non‐health fields where communities were involved in monitoring of resources or public services. Specifically, the aims were to: explore the fields where CLM or similar approaches have been used (including HIV, non‐HIV and non‐health fields) and to identify methods/tools used to monitor the effectiveness of such approaches. The synthesized findings and lessons drawn from monitoring the effectiveness of community‐led approaches in other fields can inform the development of a monitoring framework for CLM programmes.

## 2 METHODS

This scoping review used an approach adapted from Arksey and O'Malley's methodological framework for conducting scoping studies [14] and was guided by two questions:

1. Besides HIV, in what other health areas and non‐health fields are communities involved in monitoring of public services or programmes? and
2. What are the methodologies/tools used to assess the effectiveness of these programmes?

For the purposes of this study, a scoping review was defined as a type of evidence synthesis that “maps the literature on a particular topic or research area and provides an opportunity to identify key concepts; gaps in the research; and types and sources of evidence to inform practice, policymaking, and research” [15].

### 2.1 Search strategy

Three electronic databases: PubMed®, Embase® and Web of Science™ were searched from inception until 7th March 2024. The databases were selected to be comprehensive and to cover a broad range of disciplines. No limits on subject or publication type were placed on the database search. The search query consisted of variations of the term “community‐led monitoring” (see Table S1).

In addition, a web search was conducted via Google to identify relevant grey literature, including annual reports, evaluation reports, conference proceedings, news articles, working papers and webpages related to CLM programmes that fit the inclusion criterion. Search terms used included combinations of words such as: community‐led monitoring and impact/effectiveness.

Using the “snowballing” technique, references in the documents identified during the scoping review and grey literature searches were also researched if they appeared relevant to the review. On 23 June 2025, we updated our search using the same databases, search terms and inclusion criteria.

### 2.2 Citation management

All citations were imported into the web‐based bibliographic manager Rayyan [16]. Citations identified by Rayyan as duplicates were reviewed manually, and duplicate ones were removed.

### 2.3 Inclusion criteria

A two‐stage screening process was used to assess the relevance of documents identified in the search. Documents were eligible for inclusion if they described community involvement in monitoring and reporting on how services, programmes and policies were implemented and experienced at the level of communities and recipients of care [1]. As one of the aims of this review was to identify approaches to assess community monitoring of non‐HIV services, a more general inclusion criterion was adopted to include not just papers that described CLM (as typically used in HIV) but also those that described community involvement in monitoring of public services (in health and/or in other fields). Only English language publications were included.

### 2.4 Screening

The title and abstract of citations were reviewed in Rayyan by two reviewers (FM and NT). Conflicts were resolved by discussion with a third reviewer (SP). Potentially relevant citations identified proceeded to the second stage and were uploaded to reference management software Zotero. In the second stage, full‐text articles were screened for relevance.

### 2.5 Additional search for identified monitoring programmes

An additional search was used to gather more information about programmes identified during the review. Each programme name identified through the review was used as a search term in Google for associated websites, reports and/or peer‐reviewed articles, with no date restrictions on the search. The websites and documents were screened for relevance, and potentially relevant items were uploaded into Zotero for full‐text review.

### 2.6 Data charting and analysis

Once relevant documents were identified, key information was extracted about the programmes and characterized to create a database in Microsoft Excel 2010 (Microsoft Corporation, Redmond, WA). Key characteristics extracted, included: publication type, region and geographic location, field (HIV, other health, etc.), programme goals, the methods used to monitor the programme, indicators used for monitoring and the frequency with which the programme was monitored. Data were coded to identify emerging themes.

### 2.7 Consultation with stakeholders

The search phase of reviews is often supplemented with consultations to help develop key search terms, identify relevant resources, and to validate and augment review findings, providing a more nuanced, robust and useful understanding of collected data [14, 17]. In 2022, IAS—the International AIDS Society, launched a CLM programme to build a better understanding of CLM and its adoption to improve the quality and effectiveness of HIV service delivery. To guide this programme, a multi‐stakeholder CLM steering committee, including several CLM implementers, was established. This review involved consultation with the IAS CLM steering committee and staff to identify existing CLM programmes to include in the review, as well as resources to help identify other programmes.

## 3 METHODOLOGICAL REFLECTIONS

### 3.1 Terminology

The terminology used to describe the concept of community involvement in or leadership of monitoring services differed across various fields. While the term “*community‐led monitoring*” refers to the monitoring of services received by the community, not the services they themselves implement or deliver and was used predominantly in the field of HIV, and increasingly in TB, other fields used different terms. For example, “*community‐led local development*” (CLLD) was used to describe a bottom‐up approach to policy development that encourages local people to form Local Action Groups to address the social, environmental and economic challenges in their area [18].

Another approach identified was “*locally led adaptation*” (LLA), where local actors and communities lead decisions over how, when and where to adapt to the impact of climate change [19, 20]. LLA programmes are climate resilience initiatives designed, implemented and driven by local communities, institutions and stakeholders. These programmes prioritize local knowledge, context‐specific solutions and inclusive decision‐making to address climate risks [21]. LLA emphasizes shifting power and resources to the local level, enabling communities to lead in identifying their own adaptation priorities, managing climate impacts and building long‐term resilience tailored to their environmental, cultural and socio‐economic realities. In the field of natural resource conservation, the engagement of community members in monitoring and management of biodiversity and natural resources was referred to as “*locally based monitoring*” [22].

In this paper, the term “CLM” is used when communities were involved in monitoring the delivery of HIV services and the term “local monitoring approach” is used to collectively describe community involvement in monitoring of other (non‐HIV) services.

### 3.2 Assessing effectiveness

As the aim of this scoping review was to explore how the effectiveness of CLM programmes is monitored, rather than to evaluate effectiveness per se, we did not pre‐define effectiveness. We accepted the characterization of effectiveness as reported by the study authors and documented any associated monitoring approaches, indicators or outcomes used to support those claims.

### 3.3 Community‐led service delivery versus CLM

The search also identified instances where the term “community‐led” was used in the context of providing services rather than monitoring the delivery of services. One example was community‐led total sanitation (CLTS), a behaviour change approach used mainly in developing countries to improve sanitation and hygiene practices in a community. It aims to bring about spontaneous and long‐lasting behaviour change of an entire community, leading to spontaneous and long‐term abandonment of open defecation practices [23]. Many peer‐reviewed publications identified through the database search focused on CLTS. As this was not in line with the scope of this review (i.e. monitoring and reporting experiences using services), such papers were excluded.

### 3.4 Defining the extent of community involvement/leadership

Although community leadership is central to CLM, there is no uniformly agreed‐upon definition of what “leadership” in this context means. The Global Fund, for example, describes “community capacity building and leadership development” as community‐led organizations having basic legal status, functional governance, strategic plans, capacity for financial and human resource management and sufficient funding and infrastructure [24]. UNAIDS technical guidance on CLM, as well as best practices in CLM implementation, indicate that CLM programmes must be “led,” “owned” and “implemented” by directly impacted communities and users of healthcare services [12, 25]. For this review, given the general criterion adopted to include approaches from non‐HIV fields, a specific definition of “leadership” was not used as a criterion for inclusion. Any publications that were described by their authors as “community‐led” or that documented community involvement in programmes to monitor service provision/implementation were included.

## 4 RESULTS

**Figure 2:**
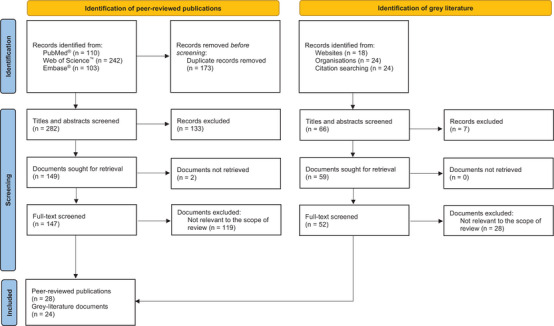
The PRISMA flow diagram for the scoping review detailing the database searches, the number of abstracts screened and the full texts retrieved.

A total of 282 unique peer‐reviewed publications (journal articles or conference abstracts) were identified through a search of PubMed®, Embase® and Web of Science™ databases, of which 147 full texts were reviewed, and 28 publications were found to be relevant. Figure 2 depicts a summary of the literature screening process. An annotated bibliography of the 28 publications identified through the review is presented in Table 1. Additionally, 24 documents were included through the search of the grey literature. Ten publications were identified in the updated search conducted on 23 June 2025 (see Table S3 for a summary of papers identified).

**Table 1:** Annotated bibliography of peer‐reviewed literature

Author (year)	Title	Domain	Type of publication	Description
Baptiste [26]	Improving HIV care in West Africa: Effects of a community treatment observatory	HIV	Peer‐reviewed conference abstract	One off evaluation based on improvements in HIV programme indicators in the International Treatment Preparedness Coalition (ITPC) established Regional Community Treatment Observatory (RCTO) in West Africa
Hoai Nguyen [27]	Community‐led quality improvement of HIV services using community scorecards in Vietnam	HIV	Peer‐reviewed conference abstract	Findings from use of Community scorecards (CSC) in two Vietnamese high‐burden provinces to reduce Stigma and Discrimination, facilitate community engagement and improve services.
Kusen [28]	Community Led Monitoring (CLM): a new CLM toolkit to generate evidence to support community‐led HIV service improvements	HIV	Peer‐reviewed conference abstract	Describes the development of Sustainable Community‐led Monitoring of HIV Services Toolkit for Key Populations the Global Fund supported Sustainability of HIV Services for Key Populations in Asia (SKPA‐2) programme
Mavasa [29]	Human rights violations against key populations in South Africa Public health facilities: findings from the Ritshidze Community‐led Monitoring program	HIV	Peer‐reviewed conference poster	One off evaluation based on survey of KP healthcare experiences in Ritshidze. Data were analysed using descriptive statistics for key service quality and human rights indicators by population and province.
Sharp [30]	Lessons learned in community‐led monitoring: early evidence from global study of the implementation landscape	HIV	Peer‐reviewed conference poster	One off evaluation via survey and interview of CLM implementers across multiple CLM approaches.
Tshuma [31]	The Transformative Impact of Community‐Led Monitoring in the South African Health System: A Comprehensive Analysis	HIV	Peer‐reviewed article	One off evaluation via interviews of CLM stakeholders.
Yawa [32]	Evaluation of Ritshidze community‐led monitoring program in South Africa	HIV	Peer‐reviewed conference poster	One off evaluation of 109 indicators grouped into 14 priority areas in Ritshidze. Longitudinal trends were calculated by the average of the quarter‐by‐quarter percentage change in each metric.
Colloff [20]	Cyclones and skinny dolphins: adaptation pathways for Pacific communities under rapid global change	Climate—community based adaptation to climate vulnerabilities	Peer‐reviewed article	This paper describes the development of a participatory MEL framework for Community‐led Adaptation Pathways in Solomon Islands (CAPSI)
Aldorfai [33]	An innovative methodology for supporting the CLLD	CLLD	Peer‐reviewed article	This study provides a method to compare regional characteristics and indicators in the EU‐based CLLD programme to monitor the development path of the regions, and also the performance of regional development programmes.
Ciampa [34]	Towards eco‐social transition: Community Regeneration Indicators respond to the polycrisis	CLLD	Peer‐reviewed article	This paper discusses the relationships between the adaptive capacity of cities and the latest participatory governance approaches. Describes community regeneration indicators including those for governance and capacity building.
Cerreta [35]	Triggering Active Communities for Cultural Creative Cities: The “Hack the City” Play ReCH Mission in the Salerno Historic Centre (Italy)	CLLD	Peer‐reviewed article	The paper describes the “Play ReCH (Re‐use Cultural Heritage)” approach, that promotes a process of collaboration, gamification and innovation in cultural heritage reuse, as an opportunity to test how cultural, creative and community‐led urban strategies can support the enhancement of heritage generating enabling environments and culturally vibrant contexts.
Pappalardo [36]	Is the partnership governance able to promote endogenous rural development? A preliminary assessment under the adaptive co‐management approach	CLLD	Peer‐reviewed article	This study proposes the Adaptive Co‐Management (ACM) theoretical framework as a model with which to study the social interactions between actors in CLLD local action groups. Includes assessment of governance principles—collective action, social learning and resilience.
Miller [37]	Using a participatory impact assessment framework to evaluate a community‐led mangrove and fisheries conservation approach in West Kalimantan, Indonesia	Conservation—marine	Peer‐reviewed article	This study presents a participatory impact assessment (PIA) framework that evaluated the outcomes of a cross‐sector community‐led conservation initiative. Community members involved in the programme identified activities and outcomes for the conservation cooperative (CC), ranking the influence of the former on the latter as well as their daily life through multiple focus group discussions (FGDs). Participants were asked to rank the impact of activities on outcomes and the scale of the outcome which was totalled to identify the most impactful programme activities and outcomes during the project period.
Davids [38]	An impact assessment tool to identify, quantify and select optimal social‐economic, ecological and health outcomes of civic environmental management interventions, in Durban South Africa	Environment	Peer‐reviewed article	Evaluates delivery of community services, environmental impact assessment (EIA) methodology to quantify the impact significance of six civic (community led) interventions implemented by the Wise Wayz Water Care (WWWC) local community programme
Danielsen [22]	The Concept, Practice, Application, and Results of Locally Based Monitoring of the Environment	Environment	Peer‐reviewed article	This article presents a summary of work to develop a theoretical and practical understanding of locally based monitoring and outlines tests of this approach in research and practice over the past 20 years.
Rana [39]	Predicting the long‐term social and ecological impacts of tree‐planting programs: Evidence from northern India	Forest conservation	Peer‐reviewed article	Most community engagement projects are of relatively short duration, which presents a major challenge to near‐term accountability as well as assessment of longer‐term social and ecological impacts. This paper addresses this challenge by identifying and empirically validating a set of predictive proxy indicators (PPIs)—measures on key variables taken during programme implementation that are predictive of longer‐term impacts—for community‐oriented tree‐planting efforts in northern India.
Eisenbarth [40]	Can community monitoring save the commons? Evidence on forest use and displacement.	Forest conservation	Peer‐reviewed article	This study employs a randomized controlled trial to investigate the impact of community‐led monitoring on forest use in 110 villages in Uganda.
Murdock [41]	Scaling the Moments That Matter® early childhood development model: how communities’ monitoring for change contributes to sustainable impact	Health—child development	Peer‐reviewed article	This paper presents a community case study of how the Moments That Matter® (MTM) Programme community‐led monitoring, evaluation and learning (MEL) system contributes to a scalable model with quality and sustainable impact.
Bedson [42]	Community engagement in outbreak response: lessons from the 2014–2016 Ebola outbreak in Sierra Leone	Health—Ebola epidemic	Peer‐reviewed article	Retrospective analysis of reports submitted by community mobilizers in the Community Led Ebola Action (CLEA) approach implemented through the Social Mobilization Action Consortium (SMAC) during the 2014–2016 Ebola epidemic in Sierra Leone.
Ogweng [43]	The effectiveness of community‐led initiatives in livestock disease control: a case of African swine fever in rural areas of Uganda	Health—livestock disease	Peer‐reviewed article	One off evaluation using semi‐structured questionnaires to selected pig farmers to establish the effectiveness of community‐led initiatives in reducing ASF outbreaks. A significant decline in the annual frequency of ASF outbreaks in both Kasawo and Namuganga sub‐counties was observed after the implementation of community‐led initiatives.
Otiso [44]	Quality improvement in the Kenyan community health system: A mechanism to drive local innovations to overcome local challenges	Health—MNCH	Peer‐reviewed conference abstract	Feasibility of implementing quality improvement approaches in community health programmes in Kenya. Impact was that CHWs extended growth monitoring from facilities to informal day‐care centres to reach more children under 5 years in Nairobi contributing to increased diagnosis of moderate acute malnutrition at community level from 1.2% to 44% cases. CHWs developed waiting cards for antenatal care to allow women who come early to be seen first in Nairobi. The average waiting time was reduced by 25 minutes per mother.
Kashyap [45]	Arogyashreni: Towards creating a replicable model for community monitoring of primary health centres in Karnataka, India	Health—primary care	Peer‐reviewed conference abstract	This conference paper describes the creation of a community‐led monitoring of primary health centres in Karnataka, India.
Bailey [51]	Multi‐level change strategies for health: learning from people‐centred advocacy in Uganda	Health—quality of service provision	Peer‐reviewed article	One off practitioner‐led analysis of programme monitoring data from 18 multi‐level health advocacy campaigns. The findings emerge capture accounts of government responses to community‐led advocacy in Accountability Can Transform Health (ACT Health) programme.
Kakade [46]	Community‐based monitoring as an accountability tool: influence on rural health services in Maharashtra, India	Health—rural	Peer‐reviewed article	One off report of results from three rounds of CBM of rural health services that took place in a period from July 2008 to December 2009 in the 220 villages spread over five pilot districts in western Maharashtra
Maksud [47]	Role of civil society action committee as a key actor for successfully implementation of bans of Tobacco Advertising, Promotion and Sponsorship (TAPS) under tobacco control laws in the southern coastal areas of Bangladesh	Health—tobacco	Peer‐reviewed conference abstract	Results from the Grambangla Unnayan Committee community‐led civil society action committees to stop Tobacco Advertising
Velásquez [48]	Making the health system work by and for Indigenous women in Guatemala: a community led multisectoral collaboration	Health systems	Peer‐reviewed article	Analysis aiming to establish the factors underlying ALIANMISAR's work, which may have contributed to its success in collaborating with other sectors to improve the provision of healthcare for Indigenous women in Guatemala. Findings come from a process of document review, key informant interviews and dialogue with a range of stakeholders at national, departmental and municipal levels.
Amollo [49]	Scaling up positive parenting practices in Uganda: research evidence from an integrated community‐led initiative for reducing violence against children in lira district, northern Uganda	Violence against children	Peer‐reviewed article	This research paper documents the use of participatory learning and action approaches in designing, implementing and monitoring interventions to prevent violence against children in post‐conflict northern Uganda.
Webb [50]	Adaptive Management of Environmental Flows	Water quality	Peer‐reviewed article	This review describes stakeholder contribution for successful adaptive management of water resources and discusses challenges in documenting and sharing lessons learnt from adaptive management.

Abbreviations: ASF, African swine fever; CHWs, community health workers; KP, key populations.

### 4.1 Communities are involved in monitoring a diverse range of public services, in health and beyond

A wide range of fields where communities are involved in monitoring public services were identified. These fields can be broadly grouped into two categories: monitoring and management of natural resources or governance of ecosystems (e.g. water quality [50], biodiversity [20, 22, 34, 37, 38, 39, 40] including conservation of marine life or forests, urban development, climate change and sustainable energy); and monitoring of health services (e.g. child development [41], epidemics [42], HIV [26, 27, 28, 29, 30, 31, 32], livestock disease [43], maternal new‐born and child health [44, 49], primary care and health systems [45, 46, 48, 51]).

The search identified seven peer‐reviewed publications related to HIV CLM [26, 27, 28, 29, 30, 31, 32], 10 from other health services [41, 42, 43, 44, 45, 46, 47, 48, 49, 51] and 11 from monitoring of natural resources [20, 22, 33, 34, 35, 36, 37, 38, 39, 40, 50].

### 4.2 Synthesis of findings from publications related to HIV CLM

The seven peer‐reviewed publications related to HIV CLM that were identified were all published in the past 5 years, between 2020 and 2024. HIV CLM programmes were focused on facility‐level acceptability, availability, affordability, adaptability and quality (AAAAQ) along the HIV treatment cascade [52], often prioritizing accessibility and quality [3, 26, 27, 28, 29, 32]. Some CLM programmes investigated instances of service denial, stigma and discrimination, and violations of human rights [3, 29, 53], which are often interlinked with AAAAQ barriers for key populations.

A common challenge reported across different HIV CLM programmes was under‐funding [30, 54], uneven, interrupted or delayed funding of CLM programmes [3, 53], funding limited to specific CLM programme elements, such as advocacy or operational research [30, 55] or lack of long‐term funding needed to implement the full CLM cycle [55].

Another challenge reported by HIV CLM programmes was around data ownership [30, 54]. The absence of clear data sharing agreements made at the exploration and planning step of the CLM programme was described as giving rise to conflicts around data ownership and accessibility [30].

The few publications that described approaches for assessing effectiveness included very few details of these approaches, either because they were conference abstracts only [26, 27, 28, 29, 30, 32] (*n* = 6) or because they focused on one aspect of the MEAL cycle and not the full cycle. All seven of the 28 peer‐reviewed publications focused on CLM in HIV described one‐off evaluations of effectiveness defined as “whether and to what extent the CLM programme achieved its goals.” There were no peer‐reviewed publications that documented results from routine evaluations of CLM programmes or described a MEAL framework for CLM.

### 4.3 Synthesis of findings from other publications

#### 4.3.1 Multistakeholder collaboration as an enabling factor

Multisectoral collaboration involves different sectors acting together to achieve outcomes that cannot be achieved by one sector alone, usually expressed in terms of shared interests. In CLM of public services, however, the shared interests might seem less obvious, because communities have the role of monitoring a key stakeholder, the government, for example, whether it be the Ministry of Health or another government department providing services. Structuring the monitoring process as a joint assessment, rather than as a community‐led inspection or audit of the government, avoids blame and helps build trust [45, 48]. Joint monitoring contributed to important improvements in health policy and legislation, health services, and infrastructure in Guatemala [48], in primary healthcare settings in India [45], and in locally based monitoring of the environment [22]. During the programme led by indigenous women in Guatemala that aimed to improve health systems, key informants from the government reported that collaboration with the community groups helped them to do their job better, achieve their goals and improve their own credibility [48]. However, it took time for this collaboration to be recognized as mutually beneficial. As Kashyap and Balasubramanium report, health staff can be apprehensive about the CLM of their health centres, but they do not always agree with the community's assessment of services [45]. In fact, when community scorecards (a two‐way, participatory, community‐led quality improvement tool) were used in Vietnam, healthcare workers gave themselves lower scores on some indicators than clients did [27].

#### 4.3.2 Challenges in sustainability of community monitoring programmes

As in HIV, the sustainability of local monitoring approaches for other programmes is also affected by a lack of funding resourced by short‐term grants from donors [22, 48]. While the voluntary nature of community participation in monitoring programmes gives credibility to their work, it also presents challenges, including unpredictability and a high turnover of volunteers [48]. Another challenge of relying exclusively on volunteers is that unpaid work is inconsistent with the principles of equity.

#### 4.3.3 Longer term impacts

Despite the limitations in sustainability of donor‐funded interventions and the challenges in demonstrating the impact of complex local monitoring approaches, evidence suggests such programmes deepen “democratic citizenship among community advocates” and can bring long‐term positive effects across different fields [22, 33, 34, 51]. Even when the impact of local monitoring programmes in improving health outcomes in the short term is not demonstrable, engagement with the political advocacy process and enhanced capacity are important to bring about transformational change in the longer term. For example, analysis of the ACT Health (Accountability Can Transform Health) programme in Uganda showed that many community advocates developed or enhanced their reputations as leaders, taking on additional advocacy issues [51]. The long‐term benefits of training and building the capacity of the community are also reflected in findings from CLLD programmes in Europe [33, 35]. Strengthening CLM can lead to indirect outcomes such as enhanced community agency, increased trust in health systems, and improved local governance through greater accountability and citizen participation in decision‐making processes.

#### 4.3.4 People‐centred monitoring and evaluation enabled scale up

Local monitoring approaches that have been scaled up have demonstrated sustainable impact through innovative, community‐led monitoring and evaluation (M&E) systems [34, 41, 51]. Experience from the Moments That Matter programme in Zambia, which has been successfully scaled up to four other countries, showed that people‐centredness and inclusivity are critical to achieving a quality, well‐functioning community‐led monitoring and evaluation system [41]. People‐centredness of M&E programmes was not just limited to health but also echoed in CLLD programmes and in programmes involving community monitoring of climate and environmental conservation. People‐centred, participatory M&E processes are based on tools that may be initially managed by external experts but can then be easily understood by local actors and used for self‐evaluation [34, 36, 41].

#### 4.3.5 Narratives, networks and numbers: an example of a participatory M&E framework

One example of a participatory M&E framework is the one by Colloff et al. for an LLA project in the Solomon Islands [20]. The framework was co‐designed with project partners to be less technical and more practical than traditional MEAL frameworks, which typically focus on quantitative data from outcomes rather than qualitative data from processes. This participatory framework allowed a nuanced and inclusive reporting structure to elucidate the key outcomes, and the co‐production and learning events that had catalysed them. The framework supplemented the quantitative assessment of traditional MEAL reporting (*numbers*) by providing a systematic way to record important events that would not typically be included in MEAL reporting. For example, this framework enabled tracking of the emergence of key insights or so‐called “ah‐ha moments” that represented breakthroughs in collective understanding of the adaptation process (*narratives*) and how processes of engagement and networking developed, including who was included or excluded (*networks*).

#### 4.3.6 From participatory M&E towards adaptive MEL

Although participatory M&E has many benefits as it incorporates local perspectives and enhances transparency and accountability, it has also been criticized for being extractive, that is depleting local actors’ time, knowledge, resources and expertise without explicitly generating value for them [21]. Coger et al. have recommended a shift towards adaptive MEL that is “locally led and context aware” [21]. This adaptive MEL is based on the following key principles:

- Recognition of and response to structural inequalities
- Promotion of local agency in decision‐making
- Understanding contextual uncertainty and complexity
- Prioritizing learning processes
- Generating value for local stakeholders.

Fundamentally, this approach recognizes that most MEAL approaches are extractive if they do not explicitly create value for the local actors. Tailoring evaluations to locally determined priorities can have the added benefit of incentivizing participation and interest in the evaluation and the adaptation intervention going forward.

## 5 DISCUSSION

As CLM gains traction as a methodology to improve the health of people from communities most affected by diseases such as HIV, TB and malaria, it is important to analyse and document the impact and share lessons learnt. Although there are several opinion pieces and review articles on the importance of engaging communities in monitoring of services [9, 41, 46] and several publications that describe how CLM or local monitoring programmes were designed [28, 30, 42, 47], few described how the effectiveness of these programmes was assessed.

There were no peer‐reviewed publications that documented results from routine evaluations or described a MEAL framework for CLM. As findings from local monitoring approaches show, having a robust M&E system is essential for improving CLM programme operations and efficiency, demonstrating impact and scaling up the interventions. Strong accountability mechanisms can contribute to sustained ownership of the CLM programme by the affected communities. Identification of areas that need improvement can foster learning, community visibility and partnerships with health facilities and decision‐makers. Using a MEAL for CLM programmes can improve services and confidence in investing in CLM and community interventions by funders and technical partners.

However, demonstrating the impact of community‐led advocacy efforts, a critical stage in the CLM cycle, is complex. Experimental and quasi‐experimental studies are seen as “rigorous,” but they often focus on short‐term outcomes [10, 51]. These approaches ignore the numerous, less linear and more complex processes through which communities utilize CLM data to advocate, secure concrete decisions and actions from decision‐makers, and to enforce the agreed decisions. Although CLM activities were reported to have contributed to short‐term gains such as improved health literacy and health‐seeking behaviour [56], absence of which consistently correlates with challenges accessing care and adverse health outcomes [57, 58], more research is needed to assess longer‐term impact that may or may not be service related (e.g. positive community engagement with health facilities, increased credibility of communities as a valuable source of information, improved monitoring skills of community members).

Monitoring of LLA programmes for climate resilience offers useful examples of impact assessments that can help inform the design of a MEAL for CLM programmes. Participatory M&E is a context‐specific, adaptable approach that engages local people directly in monitoring and evaluation processes and allows stakeholders to collectively decide how progress should be measured and how results should be acted upon [59]. MEAL for HIV can learn from these approaches.

As community‐led approaches have been applied to address a diverse range of local challenges, in health and beyond, they require flexible and adaptive approaches to not only project design and implementation but also for M&E. In practice, there is a need to gauge what does and does not work as action happens and build this into thinking and action in real time. For this reason, developmental evaluation approaches have been deemed useful in community‐led journeys as they involve a continuous and simultaneous learning process [12]. Developmental evaluation involves evaluation in parallel to project planning and implementation rather than in a sequential approach after implementation.

Measuring and monitoring the impact of community‐led initiatives, like CLD and CLM, that have a wide scope and are applied to address a range of challenges is a “best fit” process. Given each community‐led initiative has its own unique context, there is no one‐fits‐all approach, tool or a magic M&E indicator set that can be applied to all locally led initiatives. Each community initiative will need to “pick and mix” from a range of evaluative approaches, tools and measures and decide what is most relevant and useful for their local context, aspirations and budget [24].

It might seem that the purpose of CLM is to audit the government but programmes that reported successful impact indicated that all parties involved came to appreciate that its real purpose was to enable and support the health sector to do their job by pointing to the improvements in health services, and jointly developing the solutions needed to achieve the goal of wellbeing for people [10, 45, 48]. Building trust with decision‐makers and governments, although time‐consuming, can be a win‐win situation. There seems to be a growing acceptance among governments and donors that community‐led action in the form of CLM is integral to achieving health goals. However, it certainly requires stronger reinforcement and a longer‐term vision to allow CLM to become established and demonstrate impact [10, 60]. Governments can benefit from CLM as a tool for reviewing primary healthcare progress under their Universal Health Coverage plans [45] and for adapting to rapidly changing service delivery needs in the case of current and future health emergencies [10].

Long‐term sustainability of CLM programmes requires not only stable funding mechanisms but also strategies for enhancing volunteer engagement and institutionalizing CLM processes. To diversify and stabilize funding for CLM, programmes can tap into structured mechanisms such as integrating CLM within larger donor grants (e.g. Global Fund or PEPFAR funding proposals), engage public–private partnerships like EU‐style CLLD and pursue modest performance‐based stipends or cost‐sharing with local governments to reduce reliance on fragmented donor cycles. Enhancing volunteer engagement and institutionalizing CLM can be achieved by formalizing roles with clear expectations, capacity‐building, routine supportive supervision, modest stipends or performance‐based rewards, embedding volunteers in multisectoral committees (e.g. Ghana's community health management committees that support and oversee the community‐based health planning and services [61]) and using community scorecards or peer‑led data cascades to foster ownership and accountability. 

Although the search strategy for the literature review was designed to cast a wide net to identify non‐HIV fields where communities have been involved in monitoring services, it was not intended to be an exhaustive search. Therefore, there might be examples of CLM of public services that are not included in this manuscript. Since the initial search for this scoping review was conducted (in March 2024), new papers and updated programme reports have been published that document lessons learnt from implementing CLM programmes [10, 11, 62, 63]; however, none of the 10 new publications identified through the updated search in June 2025 describe a MEAL framework for CLM, so the work presented in this paper remains a novel contribution to the field (see Table S3 for a summary of new papers identified). There is progress in documenting health service improvements, but data on improving health outcomes, indirect outcomes (such as empowering community leadership) and long‐term impact of CLM programmes are lacking. These publications also acknowledge that quantifying the direct impact of CLM programmes remains challenging and requires further work [10]. Addressing this challenge will require approaches and tools for assessing the impact of CLM, as well as alignment among different stakeholders’ expectations on methodological approaches to assess the impact of CLM. Recent studies underscore the growing relevance and complexity of CLM in health systems across diverse contexts. Evidence highlights that CLM can enhance data credibility, community engagement and service accountability, particularly when embedded as a long‐term, advocacy‐driven approach [10, 64]. Key facilitators include stakeholder buy‐in, digital data tools and participatory design frameworks [63, 65, 66]. However, implementation challenges persist, such as limited political inclusion [65], funding constraints and inflexible donor structures [63], technological barriers [66] and unclear role definitions among stakeholders. Strategic recommendations emphasize building capacity, supporting data enumerators and integrating CLM into national policy frameworks to ensure sustainability and broader systemic impact [64, 67].

## 6 CONCLUSIONS

The main purpose of the scoping review was to inform the development of the MEAL framework for CLM programmes, primarily for HIV. We sought to understand where and how a MEAL framework has been applied to other health areas and non‐health fields where communities were involved in monitoring of resources or public services. Findings indicate that communities are involved in monitoring a wide range of public services; however, a MEAL framework has not been included consistently or at all as part of these monitoring programmes. Results from this scoping review capture lessons learnt and best practices from other fields to guide a way forward for MEAL frameworks for HIV CLM programmes. These findings, along with consultations with CLM implementers, have been incorporated in a guide to monitoring, evaluation, accountability and learning (MEAL) for CLM programmes to support CLM implementers in tracking progress towards outcomes and impact [68].

## COMPETING INTERESTS

The authors declare that they have no competing interests.

## AUTHORS’ CONTRIBUTIONS

FM and NT designed the review and acquired funding. FM and NT reviewed the papers. FM wrote the first draft and made subsequent revisions to the manuscript. NT and SP provided key documents and information and reviewed all drafts. All authors reviewed the first and final drafts and approved the final submission.

## FUNDING

This work was funded by IAS—the International AIDS Society through the Bill & Melinda Gates Foundation (INV‐049564).

## Supporting information




**Table S1**: Search term combinations used to identify approaches to monitor CLM programmes.
**Table S2**: Preferred Reporting Items for Systematic reviews and Meta‐Analyses extension for Scoping Reviews (PRISMA‐ScR) Checklist.
**Table S3**: Annotated bibliography of peer‐reviewed literature identified through secondary search conducted on 23 June 2025.

## Data Availability

All data generated or analysed during this study are included in this published article and its Supplementary Information Files.
