# Supplementary material for: Approaches used to monitor the effectiveness of community‐led monitoring programmes: a scoping review to inform HIV programmes
Source: J Int AIDS Soc. 2025 Jul 25;28(8):e70020. doi: 10.1002/jia2.70020 (PMC12677928; doi:10.1002/jia2.70020)
Supplement: Supplementary file 1 — Table S1: Search term combinations used to identify approaches to monitor CLM programmes. Table S2: Preferred Reporting Items for Systematic reviews and Meta‐Analyses extension for Scoping Reviews (PRISMA‐ScR) Checklist. Table S3: Annotated bibliography of peer‐reviewed literature identified through secondary search conducted on 23 June 2025. [file JIA2-28-e70020-s001.docx]

# Approaches used to monitor the effectiveness of Community-Led Monitoring (CLM) programs: a scoping review to inform HIV programmes

**Supplementary materials**

**Authors:**

Farihah Malik^1^, Nonna Turusbekova^2^, Susan Perez^3^

**Affiliations**

1. University Institute of Public Health, The University of Lahore, Lahore, Pakistan
2. Independent consultant, Antwerp, Belgium
3. AIDS Strategy Advocacy and Policy, Hanoi, Vietnam

**Corresponding author:**

Dr. Farihah Malik (farihah.malik@pht.uol.edu.pk)

# S1 Search term combinations used to identify approaches to monitor CLM programs

| **Database** | **Platform** | **Search Terms / Strategy** | **Date of Search** | **Limits Applied** |
| --- | --- | --- | --- | --- |
| PubMed | NCBI | ("community-led monitoring"[Title/Abstract] OR "community led monitoring"[Title/Abstract]) | 07 March 2024 | None |
| Web of Science | Clarivate | TS=("community-led monitoring") | 07 March 2024 | None |
| Embase | Elsevier | #1 'community led monitoring'/exp OR 'community led monitoring' #2 'community led'/exp AND monitoring/exp Final: #1 AND #2 | 07 March 2024 | None |
| Google Search | Google | “Impact of community led monitoring”  “Impact of community led monitoring of public services”  “Effectiveness of community led monitoring”  “Impact of community based monitoring”  “Effectiveness of community based monitoring” | 07 March 2024 | First 3 pages of results screened |

**Notes:**

- No language or date restrictions were applied
- On 23 June 2025, we updated our search using the same databases, search terms, and inclusion criteria.

**S2 Preferred Reporting Items for Systematic reviews and Meta-Analyses extension for Scoping Reviews (PRISMA-ScR) Checklist**

| **SECTION** | **ITEM** | **PRISMA-ScR CHECKLIST ITEM** | **REPORTED ON PAGE #** |
| --- | --- | --- | --- |
| **TITLE** | | | |
| Title | 1 | Identify the report as a scoping review. | 1 |
| **ABSTRACT** | | | |
| Structured summary | 2 | Provide a structured summary that includes (as applicable): background, objectives, eligibility criteria, sources of evidence, charting methods, results, and conclusions that relate to the review questions and objectives. | 2 |
| **INTRODUCTION** | | | |
| Rationale | 3 | Describe the rationale for the review in the context of what is already known. Explain why the review questions/objectives lend themselves to a scoping review approach. | 4-5 |
| Objectives | 4 | Provide an explicit statement of the questions and objectives being addressed with reference to their key elements (e.g., population or participants, concepts, and context) or other relevant key elements used to conceptualize the review questions and/or objectives. | 5 |
| **METHODS** | | | |
| Protocol and registration | 5 | Indicate whether a review protocol exists; state if and where it can be accessed (e.g., a Web address); and if available, provide registration information, including the registration number. | Not applicable |
| Eligibility criteria | 6 | Specify characteristics of the sources of evidence used as eligibility criteria (e.g., years considered, language, and publication status), and provide a rationale. | 7-8 |
| Information sources* | 7 | Describe all information sources in the search (e.g., databases with dates of coverage and contact with authors to identify additional sources), as well as the date the most recent search was executed. | 7 |
| Search | 8 | Present the full electronic search strategy for at least 1 database, including any limits used, such that it could be repeated. | Supplementary materials |
| Selection of sources of evidence† | 9 | State the process for selecting sources of evidence (i.e., screening and eligibility) included in the scoping review. | Not applicable |
| Data charting process‡ | 10 | Describe the methods of charting data from the included sources of evidence (e.g., calibrated forms or forms that have been tested by the team before their use, and whether data charting was done independently or in duplicate) and any processes for obtaining and confirming data from investigators. | 9 |
| Data items | 11 | List and define all variables for which data were sought and any assumptions and simplifications made. | 9 |
| Critical appraisal of individual sources of evidence§ | 12 | If done, provide a rationale for conducting a critical appraisal of included sources of evidence; describe the methods used and how this information was used in any data synthesis (if appropriate). | Not applicable |
| Synthesis of results | 13 | Describe the methods of handling and summarizing the data that were charted. | 9-11 |
| **RESULTS** | | | |
| Selection of sources of evidence | 14 | Give numbers of sources of evidence screened, assessed for eligibility, and included in the review, with reasons for exclusions at each stage, ideally using a flow diagram. | 12 |
| Characteristics of sources of evidence | 15 | For each source of evidence, present characteristics for which data were charted and provide the citations. | Table 1 |
| Critical appraisal within sources of evidence | 16 | If done, present data on critical appraisal of included sources of evidence (see item 12). | Not applicable |
| Results of individual sources of evidence | 17 | For each included source of evidence, present the relevant data that were charted that relate to the review questions and objectives. | Table 1 |
| Synthesis of results | 18 | Summarize and/or present the charting results as they relate to the review questions and objectives. | 31-35 |
| **DISCUSSION** | | | |
| Summary of evidence | 19 | Summarize the main results (including an overview of concepts, themes, and types of evidence available), link to the review questions and objectives, and consider the relevance to key groups. | 36-37 |
| Limitations | 20 | Discuss the limitations of the scoping review process. | 39 |
| Conclusions | 21 | Provide a general interpretation of the results with respect to the review questions and objectives, as well as potential implications and/or next steps. | 40 |
| **FUNDING** | | | |
| Funding | 22 | Describe sources of funding for the included sources of evidence, as well as sources of funding for the scoping review. Describe the role of the funders of the scoping review. | 41 |

JBI = Joanna Briggs Institute; PRISMA-ScR = Preferred Reporting Items for Systematic reviews and Meta-Analyses extension for Scoping Reviews.

* Where *sources of evidence* (see second footnote) are compiled from, such as bibliographic databases, social media platforms, and Web sites.

† A more inclusive/heterogeneous term used to account for the different types of evidence or data sources (e.g., quantitative and/or qualitative research, expert opinion, and policy documents) that may be eligible in a scoping review as opposed to only studies. This is not to be confused with *information sources* (see first footnote).

‡ The frameworks by Arksey and O’Malley (6) and Levac and colleagues (7) and the JBI guidance (4, 5) refer to the process of data extraction in a scoping review as data charting*.*

§ The process of systematically examining research evidence to assess its validity, results, and relevance before using it to inform a decision. This term is used for items 12 and 19 instead of "risk of bias" (which is more applicable to systematic reviews of interventions) to include and acknowledge the various sources of evidence that may be used in a scoping review (e.g., quantitative and/or qualitative research, expert opinion, and policy document).

*From:* Tricco AC, Lillie E, Zarin W, O'Brien KK, Colquhoun H, Levac D, et al. PRISMA Extension for Scoping Reviews (PRISMAScR): Checklist and Explanation. Ann Intern Med. 2018;169:467–473. [doi: 10.7326/M18-0850](http://annals.org/aim/fullarticle/2700389/prisma-extension-scoping-reviews-prisma-scr-checklist-explanation).

**S 3 Annotated bibliography of peer-reviewed literature identified through secondary search conducted on 23 June 2025**

| **Author (Year)** | **Title** | **Domain** | **Type of publication** | **Description** |
| --- | --- | --- | --- | --- |
| Chorna 2025 | Perspectives on multisectoral accountability framework to end tuberculosis in the Eastern Europe and Central Asia region: a mixed-methods study | TB | Peer-reviewed article | A mixed-methods community-based study. Surveys, interviews, and focus groups were conducted online with TB-engaged community and civil society representatives in Belarus, Kazakhstan, Moldova, Tajikistan, and Ukraine from January to June 2021.  “few indicated inclusion in … political and program impact of community-led monitoring (n = 16, 30·2%)” |
| Chan 2025 | Classifying strategies for building community health movements: a guide for implementers | Health systems | Peer-reviewed article | A rapid review to synthesise evidence on how community-led health initiatives can contribute to reducing the burdens on the health care system.  Focused on community movements or affiliated constructs in upper-middle and high-income Asian countries, conducted between 2014 and 2021.  The MovEMENTs checklist and related strategies were elicited through the review. The six Intermediate Results include to: (1) Move the community to be recruited and retained (2) Engage capacity and build capability; (3) Maintain emotional resonance; (4) Embed participatory approaches; (5) Nurture network building and partnerships; (6) Team up to improve commissioning and funding structures. |
| Tamayo 2024 | Stakeholder Perceptions towards a Mobile Application for Community-Led Monitoring of Tuberculosis Services in Metro Manila, Philippines: A Qualitative Study | TB | Peer-reviewed article | Qualitative research to determine the perceptions of people with TB and health workers on community LED monitoring of TB services.  The community-led monitoring mobile application was shown to be acceptable both to TB healthcare providers and patients. It enhances information access and streamlines the process of reporting care barriers. The application also allows persons with TB to interact with one another, potentially eliminating stigma and discrimination. Potential challenges to implementing the CLM program include issues with internet connectivity, costs, and human resources. |
| Sharp 2024 | Facilitators and barriers to community-led monitoring of health programs: Qualitative evidence from the global implementation landscape | HIV | Peer-reviewed article | (An abstract from the study was included in our review before this paper was published.)  Twenty-five CLM implementors representing 21 countries participated in an interview. Early generation of buy-in from diverse stakeholders was noted as critical for CLM success. Leveraging existing networks of service users and community organizations to implement CLM also helped to maximize program reach and resources. Uncertainty around CLM's purpose and roles among CLM stakeholders resulted in challenges to community leadership and ownership of programs. Respondents also described challenges with underfunded programs, especially advocacy components, and inflexible donor funding mechanisms. Critical capacity gaps remain around advocacy and electronic data collection and use. |
| Rambau 2024 | Power, data and social accountability: defining a community-led monitoring model for strengthened health service delivery | HIV | Peer-reviewed article | Opinion piece proposing a set of unifying principles for CLM to support the impact on the quality and availability of health services.  CLM efforts should represent a broad and collective community response, led independently by impacted communities, incorporating both data collection and advocacy, and should be understood as a long-term approach to building meaningful engagement in systems-wide improvements rather than discrete interventions. |
| Lauer 2024 | Defining community-led monitoring and its role in programme-embedded learning: lessons from the Citizen Science Project in Malawi and South Africa | HIV | Peer-reviewed article | This commentary examines a CLM initiative in Malawi and South Africa to highlight the crucial role of CLM in bolstering the Programme Science framework.  The Citizen Science Project implements CLM continuously at 33 health facilities: 14 in Malawi and 19 in South Africa.  While quantifying direct impact remains challenging due to the project's design, CLM proves to be a robust methodology that generates credible data and produces impactful outcomes. |
| Khwairakpam 2025 | Community-led monitoring of HIV and viral hepatitis services: lessons learned and impacts from India and Indonesia | HIV | Peer-reviewed article | CBOs are monitoring 12 health facilities from local districts to referral hospitals that provide public services for HIV and viral hepatitis. CLM indicators are based on national guidelines and policies and target essential components of the AAAQ framework covering HIV, hepatitis B (HBV) and hepatitis C (HCV).  This article describes how community members identified what they wanted to monitor and designed a monitoring framework, collected data through our digital data collection application and advocated for change in health facility operations. |
| Hargreaves 2024 | Programme science methodologies and practices that address “FURRIE” challenges: examples from the field | HIV | Peer-reviewed article | This paper discusses selected HIV/STI (sexually transmitted infection) programme science case studies to illustrate how programme science rises to the FURRIE challenges. |
| Goslett 2025 | Time to be seen and heard: Including children’s and adolescents’ voices in the South African TB response | TB | Peer-reviewed article | Advocates for inclusion of children’s feedback in community-led monitoring efforts. |
| Casella 2024 | Strengthening person-centered care through quality improvement: a mixed-methods study examining implementation of the Person-Centered Care Assessment Tool in Zambian health facilities | HIV | Peer-reviewed article | This study team developed the Person-Centered Care Assessment Tool (PCC-AT), which measures PCC service delivery within HIV treatment settings.  A higher proportion of urban and peri-urban compared to rural facilities identified actions related to community-led monitoring. |
